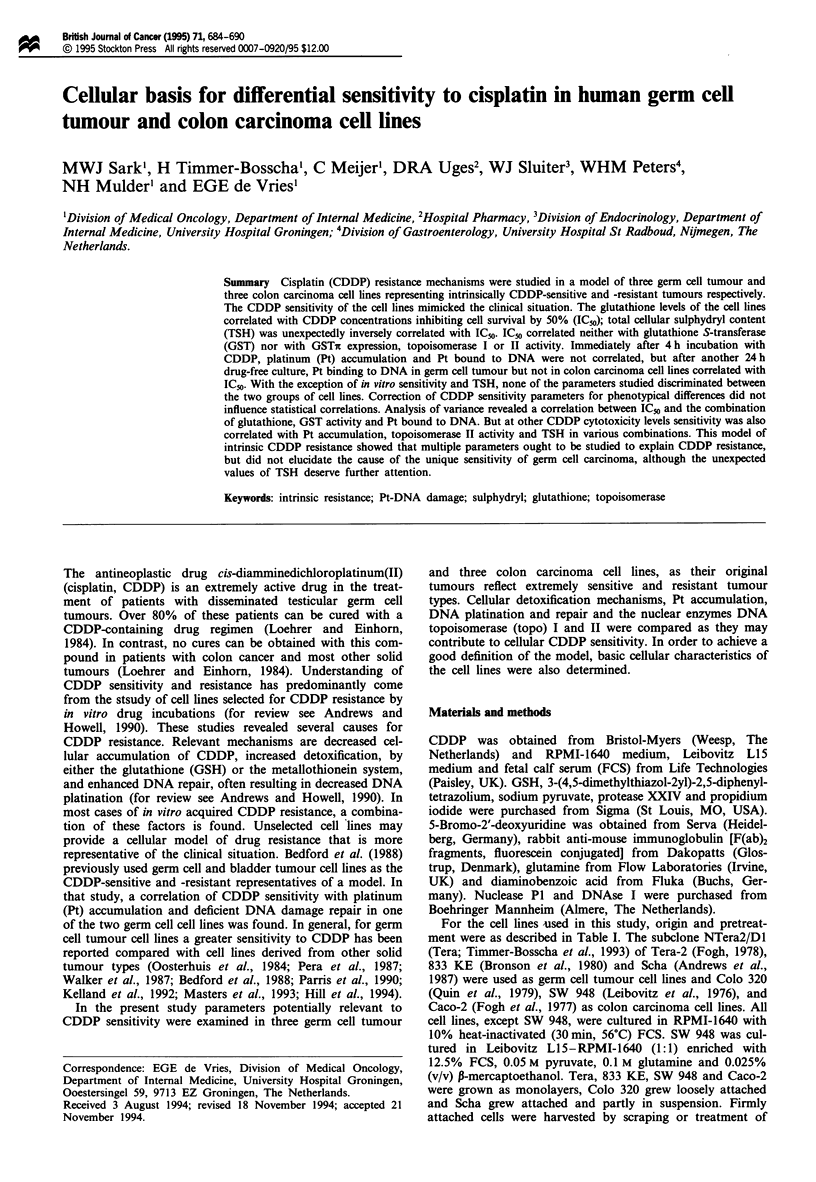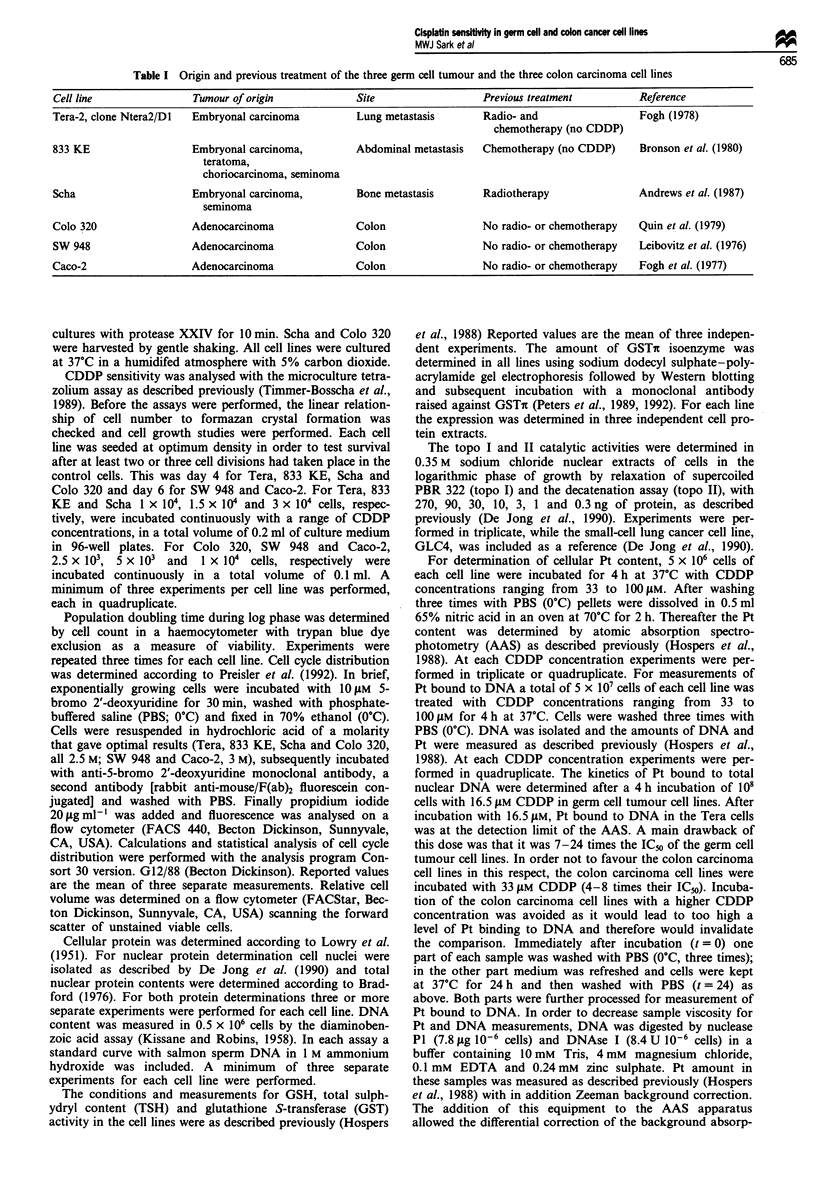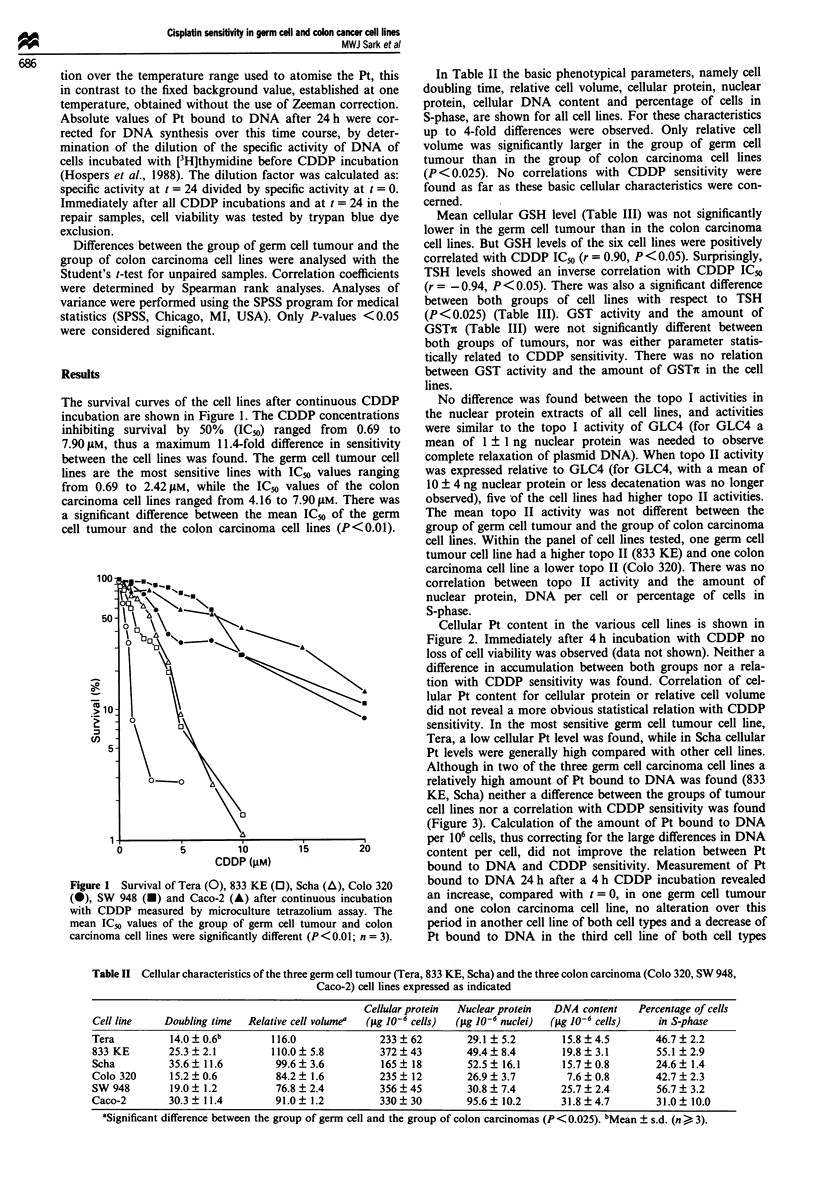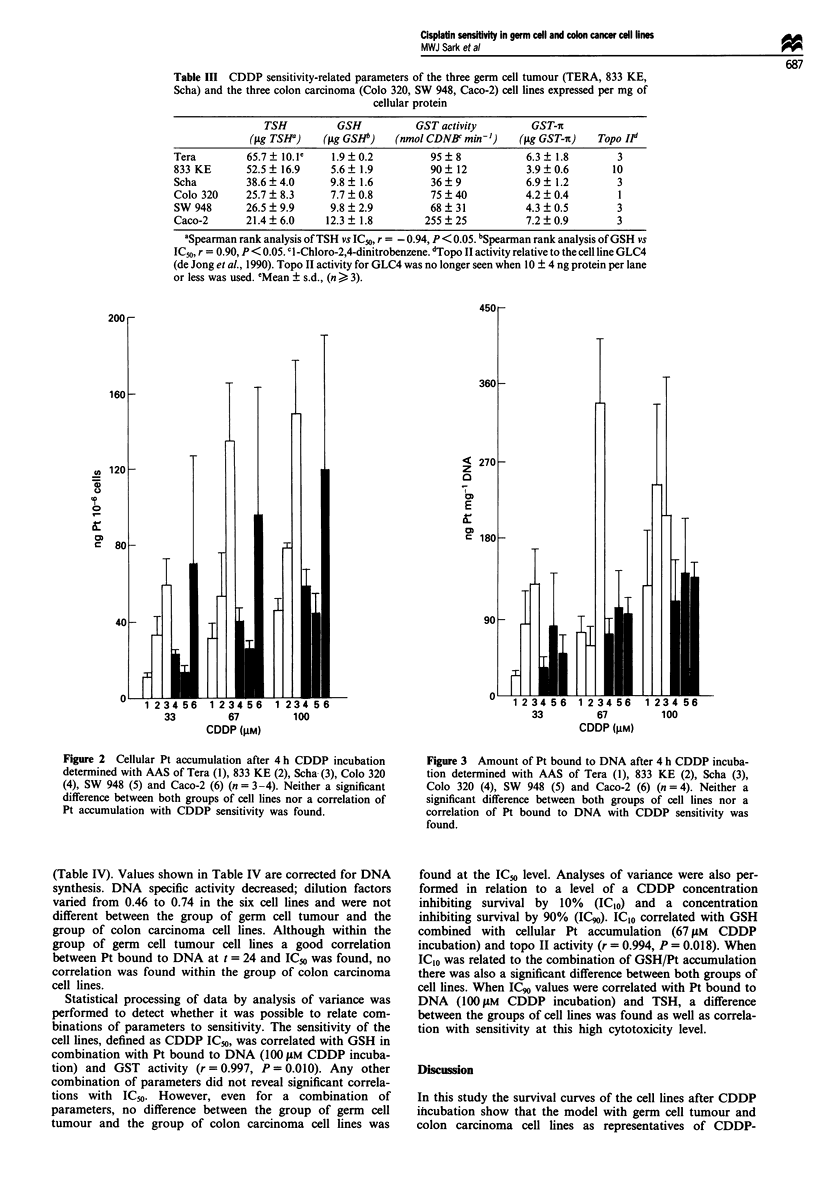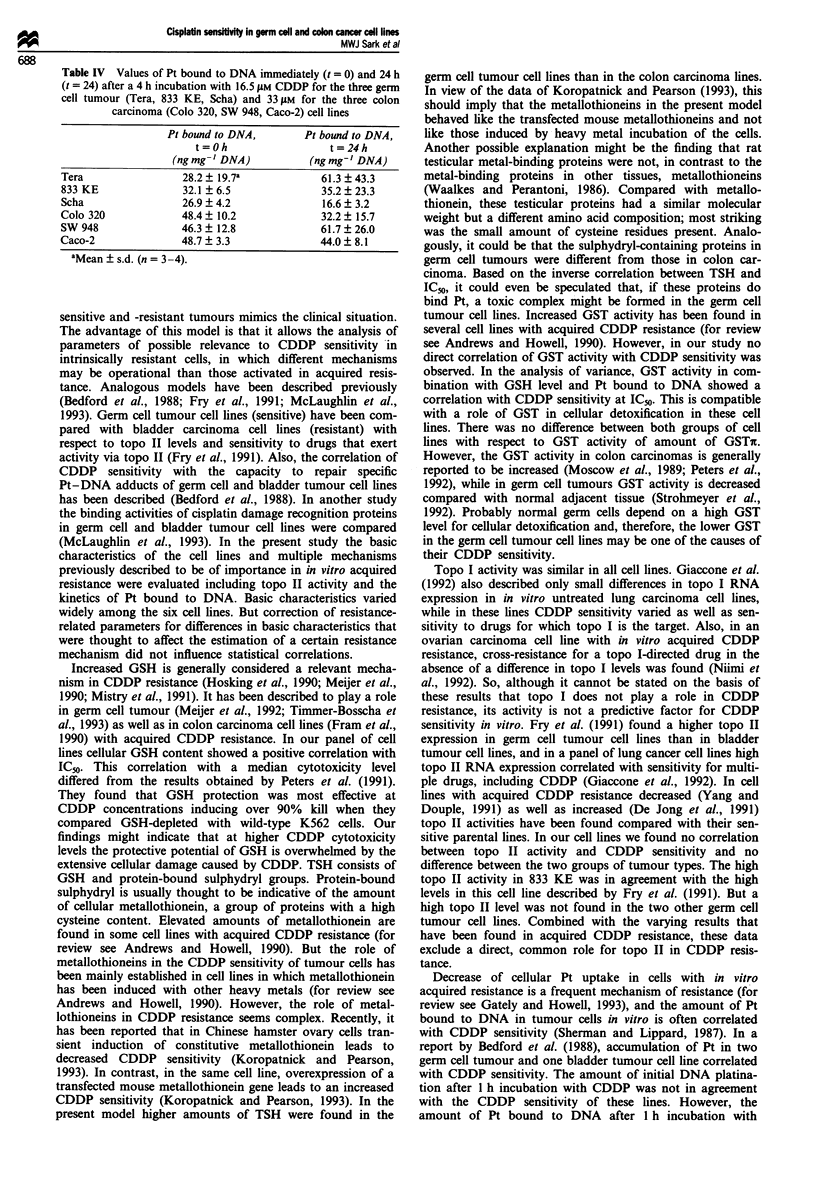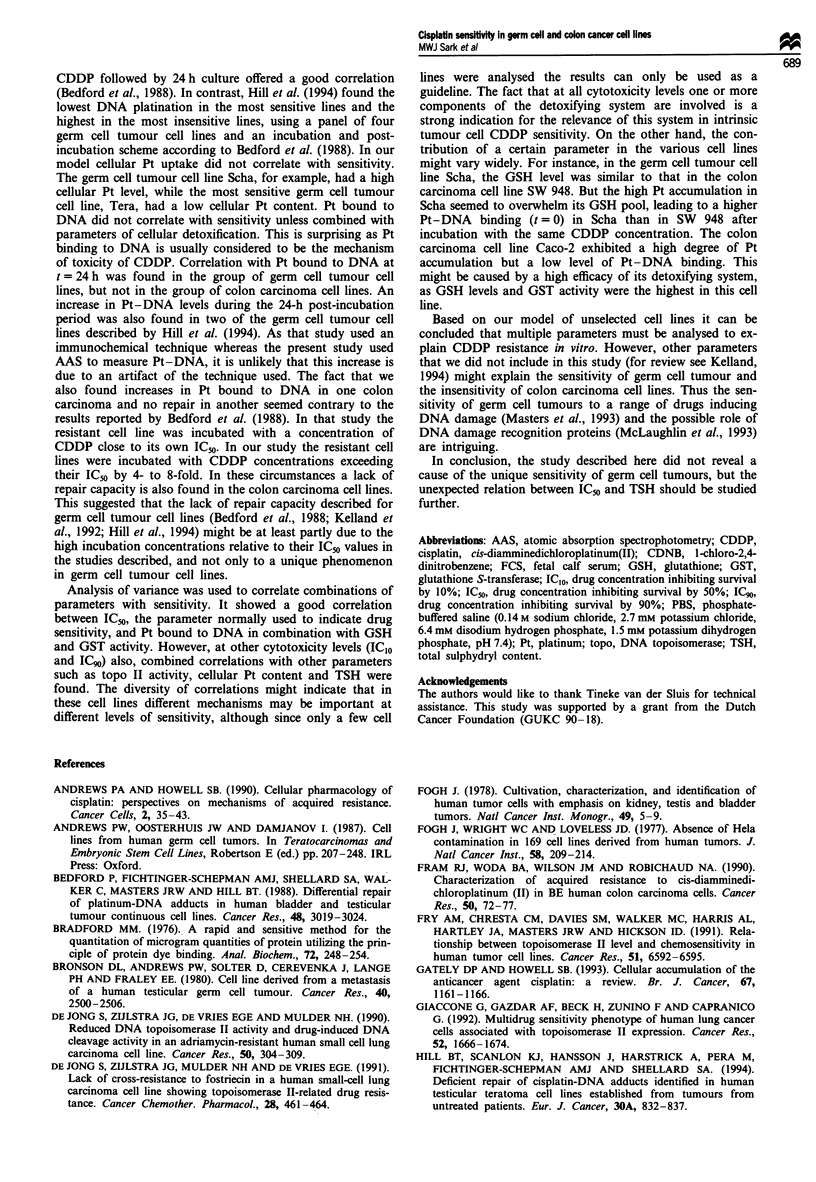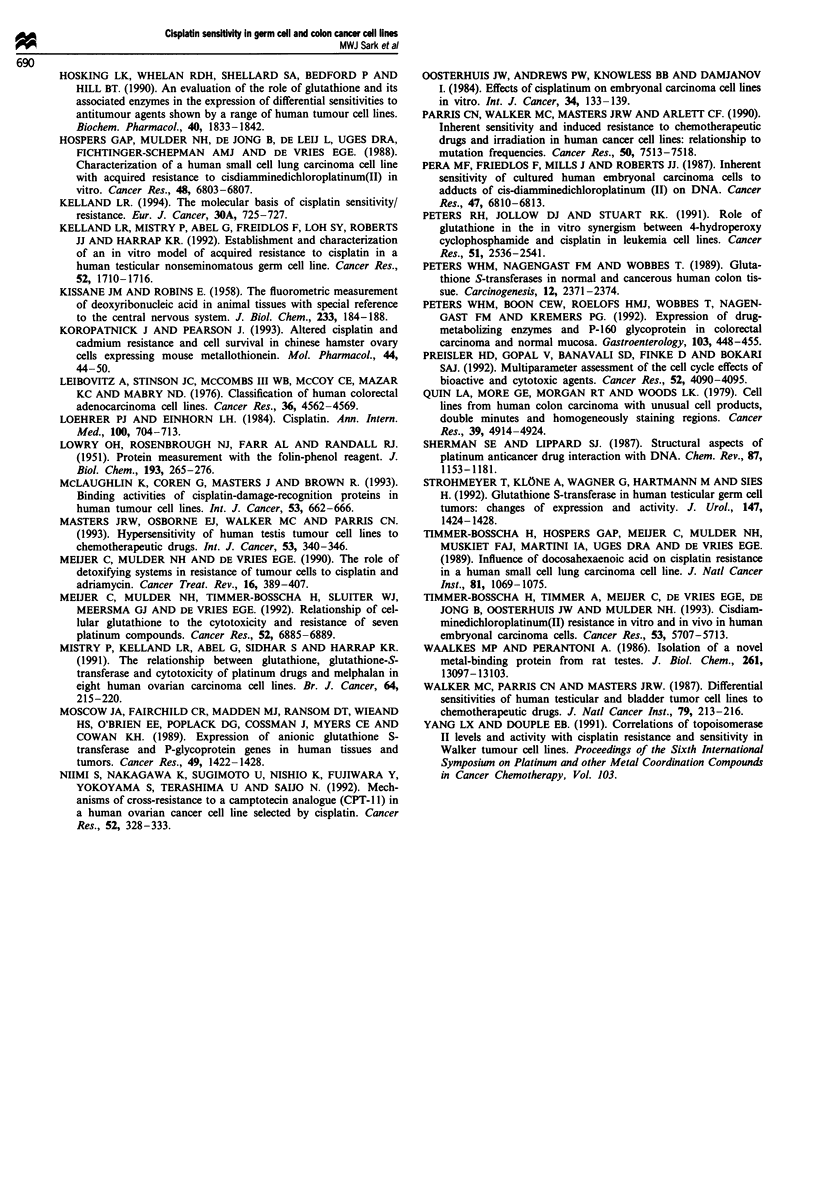# Cellular basis for differential sensitivity to cisplatin in human germ cell tumour and colon carcinoma cell lines.

**DOI:** 10.1038/bjc.1995.135

**Published:** 1995-04

**Authors:** M. W. Sark, H. Timmer-Bosscha, C. Meijer, D. R. Uges, W. J. Sluiter, W. H. Peters, N. H. Mulder, E. G. de Vries

**Affiliations:** Department of Internal Medicine, University Hospital Groningen, The Netherlands.

## Abstract

Cisplatin (CDDP) resistance mechanisms were studied in a model of three germ cell tumour and three colon carcinoma cell lines representing intrinsically CDDP-sensitive and -resistant tumours respectively. The CDDP sensitivity of the cell lines mimicked the clinical situation. The glutathione levels of the cell lines correlated with CDDP concentrations inhibiting cell survival by 50% (IC50); total cellular sulphydryl content (TSH) was unexpectedly inversely correlated with IC50. IC50 correlated neither with glutathione S-transferase (GST) nor with GST pi expression, topoisomerase I or II activity. Immediately after 4 h incubation with CDDP, platinum (Pt) accumulation and Pt bound to DNA were not correlated, but after another 24 h drug-free culture, Pt binding to DNA in germ cell tumour but not in colon carcinoma cell lines correlated with IC50. With the exception of in vitro sensitivity and TSH, none of the parameters studied discriminated between the two groups of cell lines. Correction of CDDP sensitivity parameters for phenotypical differences did not influence statistical correlations. Analysis of variance revealed a correlation between IC50 and the combination of glutathione, GST activity and Pt bound to DNA. But at other CDDP cytotoxicity levels sensitivity was also correlated with Pt accumulation, topoisomerase II activity and TSH in various combinations. This model of intrinsic CDDP resistance showed that multiple parameters ought to be studied to explain CDDP resistance, but did not elucidate the cause of the unique sensitivity of germ cell carcinoma, although the unexpected values of TSH deserve further attention.


British Journal of Cancer (1995) 71, 684-690
© 1995 Stockton Press All rights reserved 0007-0920/95 $12.00

### Cellular basis for differential sensitivity to cisplatin in human germ cell tumour and colon carcinoma cell lines

MWJ Sark[1], H Timmer-Bosscha[1], C Meijer[1], DRA Uges[2], WJ Sluiter[3], WHM Peters[4], NH Mulder[1] and EGE de Vries[1]

[1]*Division of Medical Oncology, Department of Internal Medicine,* [2]*Hospital Pharmacy,* [3]*Division of Endocrinology, Department of Internal Medicine, University Hospital Groningen;* [4]*Division of Gastroenterology, University Hospital St Radboud, Nijmegen, The Netherlands.*

**Summary** Cisplatin (CDDP) resistance mechanisms were studied in a model of three germ cell tumour and three colon carcinoma cell lines representing intrinsically CDDP-sensitive and -resistant tumours respectively. The CDDP sensitivity of the cell lines mimicked the clinical situation. The glutathione levels of the cell lines correlated with CDDP concentrations inhibiting cell survival by 50% ($IC_{50}$); total cellular sulphydryl content (TSH) was unexpectedly inversely correlated with $IC_{50}$. $IC_{50}$ correlated neither with glutathione *S*-transferase (GST) nor with GST$\pi$ expression, topoisomerase I or II activity. Immediately after 4 h incubation with CDDP, platinum (Pt) accumulation and Pt bound to DNA were not correlated, but after another 24 h drug-free culture, Pt binding to DNA in germ cell tumour but not in colon carcinoma cell lines correlated with $IC_{50}$. With the exception of *in vitro* sensitivity and TSH, none of the parameters studied discriminated between the two groups of cell lines. Correction of CDDP sensitivity parameters for phenotypical differences did not influence statistical correlations. Analysis of variance revealed a correlation between $IC_{50}$ and the combination of glutathione, GST activity and Pt bound to DNA. But at other CDDP cytotoxicity levels sensitivity was also correlated with Pt accumulation, topoisomerase II activity and TSH in various combinations. This model of intrinsic CDDP resistance showed that multiple parameters ought to be studied to explain CDDP resistance, but did not elucidate the cause of the unique sensitivity of germ cell carcinoma, although the unexpected values of TSH deserve further attention.

**Keywords**: intrinsic resistance; Pt-DNA damage; sulphydryl; glutathione; topoisomerase

The antineoplastic drug *cis*-diamminedichloroplatinum(II) (cisplatin, CDDP) is an extremely active drug in the treatment of patients with disseminated testicular germ cell tumours. Over 80% of these patients can be cured with a CDDP-containing drug regimen (Loehrer and Einhorn, 1984). In contrast, no cures can be obtained with this compound in patients with colon cancer and most other solid tumours (Loehrer and Einhorn, 1984). Understanding of CDDP sensitivity and resistance has predominantly come from the stsudy of cell lines selected for CDDP resistance by *in vitro* drug incubations (for review see Andrews and Howell, 1990). These studies revealed several causes for CDDP resistance. Relevant mechanisms are decreased cellular accumulation of CDDP, increased detoxification, by either the glutathione (GSH) or the metallothionein system, and enhanced DNA repair, often resulting in decreased DNA platination (for review see Andrews and Howell, 1990). In most cases of *in vitro* acquired CDDP resistance, a combination of these factors is found. Unselected cell lines may provide a cellular model of drug resistance that is more representative of the clinical situation. Bedford *et al.* (1988) previously used germ cell and bladder tumour cell lines as the CDDP-sensitive and -resistant representatives of a model. In that study, a correlation of CDDP sensitivity with platinum (Pt) accumulation and deficient DNA damage repair in one of the two germ cell cell lines was found. In general, for germ cell tumour cell lines a greater sensitivity to CDDP has been reported compared with cell lines derived from other solid tumour types (Oosterhuis *et al.*, 1984; Pera *et al.*, 1987; Walker *et al.*, 1987; Bedford *et al.*, 1988; Parris *et al.*, 1990; Kelland *et al.*, 1992; Masters *et al.*, 1993; Hill *et al.*, 1994).

In the present study parameters potentially relevant to CDDP sensitivity were examined in three germ cell tumour and three colon carcinoma cell lines, as their original tumours reflect extremely sensitive and resistant tumour types. Cellular detoxification mechanisms, Pt accumulation, DNA platination and repair and the nuclear enzymes DNA topoisomerase (topo) I and II were compared as they may contribute to cellular CDDP sensitivity. In order to achieve a good definition of the model, basic cellular characteristics of the cell lines were also determined.

Correspondence: EGE de Vries, Division of Medical Oncology, Department of Internal Medicine, University Hospital Groningen, Oeestersingel 59, 9713 EZ Groningen, The Netherlands.
Received 3 August 1994; revised 18 November 1994; accepted 21 November 1994.

### Materials and methods

CDDP was obtained from Bristol-Myers (Weesp, The Netherlands) and RPMI-1640 medium, Leibovitz L15 medium and fetal calf serum (FCS) from Life Technologies (Paisley, UK). GSH, 3-(4,5-dimethylthiazol-2yl)-2,5-diphenyltetrazolium, sodium pyruvate, protease XXIV and propidium iodide were purchased from Sigma (St Louis, MO, USA). 5-Bromo-2′-deoxyuridine was obtained from Serva (Heidelberg, Germany), rabbit anti-mouse immunoglobulin [$F(ab)_2$ fragments, fluorescein conjugated] from Dakopatts (Glostrup, Denmark), glutamine from Flow Laboratories (Irvine, UK) and diaminobenzoic acid from Fluka (Buchs, Germany). Nuclease P1 and DNAse I were purchased from Boehringer Mannheim (Almere, The Netherlands).

For the cell lines used in this study, origin and pretreatment were as described in Table I. The subclone NTera2/D1 (Tera; Timmer-Bosscha *et al.*, 1993) of Tera-2 (Fogh, 1978), 833 KE (Bronson *et al.*, 1980) and Scha (Andrews *et al.*, 1987) were used as germ cell tumour cell lines and Colo 320 (Quin *et al.*, 1979), SW 948 (Leibovitz *et al.*, 1976), and Caco-2 (Fogh *et al.*, 1977) as colon carcinoma cell lines. All cell lines, except SW 948, were cultured in RPMI-1640 with 10% heat-inactivated (30 min, 56°C) FCS. SW 948 was cultured in Leibovitz L15-RPMI-1640 (1:1) enriched with 12.5% FCS, 0.05 M pyruvate, 0.1 M glutamine and 0.025% (v/v) $\beta$-mercaptoethanol. Tera, 833 KE, SW 948 and Caco-2 were grown as monolayers, Colo 320 grew loosely attached and Scha grew attached and partly in suspension. Firmly attached cells were harvested by scraping or treatment of

**Table I** Origin and previous treatment of the three germ cell tumour and the three colon carcinoma cell lines

| *Cell line* | *Tumour of origin* | *Site* | *Previous treatment* | *Reference* |
|---|---|---|---|---|
| Tera-2, clone Ntera2/D1 | Embryonal carcinoma | Lung metastasis | Radio- and chemotherapy (no CDDP) | Fogh (1978) |
| 833 KE | Embryonal carcinoma, teratoma, choriocarcinoma, seminoma | Abdominal metastasis | Chemotherapy (no CDDP) | Bronson *et al.* (1980) |
| Scha | Embryonal carcinoma, seminoma | Bone metastasis | Radiotherapy | Andrews *et al.* (1987) |
| Colo 320 | Adenocarcinoma | Colon | No radio- or chemotherapy | Quin *et al.* (1979) |
| SW 948 | Adenocarcinoma | Colon | No radio- or chemotherapy | Leibovitz *et al.* (1976) |
| Caco-2 | Adenocarcinoma | Colon | No radio- or chemotherapy | Fogh *et al.* (1977) |

cultures with protease XXIV for 10 min. Scha and Colo 320 were harvested by gentle shaking. All cell lines were cultured at 37°C in a humidifed atmosphere with 5% carbon dioxide.

CDDP sensitivity was analysed with the microculture tetrazolium assay as described previously (Timmer-Bosscha *et al.*, 1989). Before the assays were performed, the linear relationship of cell number to formazan crystal formation was checked and cell growth studies were performed. Each cell line was seeded at optimum density in order to test survival after at least two or three cell divisions had taken place in the control cells. This was day 4 for Tera, 833 KE, Scha and Colo 320 and day 6 for SW 948 and Caco-2. For Tera, 833 KE and Scha $1 \times 10^4$, $1.5 \times 10^4$ and $3 \times 10^4$ cells, respectively, were incubated continuously with a range of CDDP concentrations, in a total volume of 0.2 ml of culture medium in 96-well plates. For Colo 320, SW 948 and Caco-2, $2.5 \times 10^3$, $5 \times 10^3$ and $1 \times 10^4$ cells, respectively were incubated continuously in a total volume of 0.1 ml. A minimum of three experiments per cell line was performed, each in quadruplicate.

Population doubling time during log phase was determined by cell count in a haemocytometer with trypan blue dye exclusion as a measure of viability. Experiments were repeated three times for each cell line. Cell cycle distribution was determined according to Preisler *et al.* (1992). In brief, exponentially growing cells were incubated with 10 μM 5-bromo 2′-deoxyuridine for 30 min, washed with phosphate-buffered saline (PBS; 0°C) and fixed in 70% ethanol (0°C). Cells were resuspended in hydrochloric acid of a molarity that gave optimal results (Tera, 833 KE, Scha and Colo 320, all 2.5 M; SW 948 and Caco-2, 3 M), subsequently incubated with anti-5-bromo 2′-deoxyuridine monoclonal antibody, a second antibody [rabbit anti-mouse/$F(ab)_2$ fluorescein conjugated] and washed with PBS. Finally propidium iodide 20 μg $ml^{-1}$ was added and fluorescence was analysed on a flow cytometer (FACS 440, Becton Dickinson, Sunnyvale, CA, USA). Calculations and statistical analysis of cell cycle distribution were performed with the analysis program Consort 30 version. G12/88 (Becton Dickinson). Reported values are the mean of three separate measurements. Relative cell volume was determined on a flow cytometer (FACStar, Becton Dickinson, Sunnyvale, CA, USA) scanning the forward scatter of unstained viable cells.

Cellular protein was determined according to Lowry *et al.* (1951). For nuclear protein determination cell nuclei were isolated as described by De Jong *et al.* (1990) and total nuclear protein contents were determined according to Bradford (1976). For both protein determinations three or more separate experiments were performed for each cell line. DNA content was measured in $0.5 \times 10^6$ cells by the diaminobenzoic acid assay (Kissane and Robins, 1958). In each assay a standard curve with salmon sperm DNA in 1 M ammonium hydroxide was included. A minimum of three separate experiments for each cell line were performed.

The conditions and measurements for GSH, total sulphydryl content (TSH) and glutathione *S*-transferase (GST) activity in the cell lines were as described previously (Hospers *et al.*, 1988) Reported values are the mean of three independent experiments. The amount of GSTπ isoenzyme was determined in all lines using sodium dodecyl sulphate–polyacrylamide gel electrophoresis followed by Western blotting and subsequent incubation with a monoclonal antibody raised against GSTπ (Peters *et al.*, 1989, 1992). For each line the expression was determined in three independent cell protein extracts.

The topo I and II catalytic activities were determined in 0.35 M sodium chloride nuclear extracts of cells in the logarithmic phase of growth by relaxation of supercoiled PBR 322 (topo I) and the decatenation assay (topo II), with 270, 90, 30, 10, 3, 1 and 0.3 ng of protein, as described previously (De Jong *et al.*, 1990). Experiments were performed in triplicate, while the small-cell lung cancer cell line, GLC4, was included as a reference (De Jong *et al.*, 1990).

For determination of cellular Pt content, $5 \times 10^6$ cells of each cell line were incubated for 4 h at 37°C with CDDP concentrations ranging from 33 to 100 μM. After washing three times with PBS (0°C) pellets were dissolved in 0.5 ml 65% nitric acid in an oven at 70°C for 2 h. Thereafter the Pt content was determined by atomic absorption spectrophotometry (AAS) as described previously (Hospers *et al.*, 1988). At each CDDP concentration experiments were performed in triplicate or quadruplicate. For measurements of Pt bound to DNA a total of $5 \times 10^7$ cells of each cell line was treated with CDDP concentrations ranging from 33 to 100 μM for 4 h at 37°C. Cells were washed three times with PBS (0°C). DNA was isolated and the amounts of DNA and Pt were measured as described previously (Hospers *et al.*, 1988). At each CDDP concentration experiments were performed in quadruplicate. The kinetics of Pt bound to total nuclear DNA were determined after a 4 h incubation of $10^8$ cells with 16.5 μM CDDP in germ cell tumour cell lines. After incubation with 16.5 μM, Pt bound to DNA in the Tera cells was at the detection limit of the AAS. A main drawback of this dose was that it was 7–24 times the $IC_{50}$ of the germ cell tumour cell lines. In order not to favour the colon carcinoma cell lines in this respect, the colon carcinoma cell lines were incubated with 33 μM CDDP (4–8 times their $IC_{50}$). Incubation of the colon carcinoma cell lines with a higher CDDP concentration was avoided as it would lead to too high a level of Pt binding to DNA and therefore would invalidate the comparison. Immediately after incubation ($t = 0$) one part of each sample was washed with PBS (0°C, three times); in the other part medium was refreshed and cells were kept at 37°C for 24 h and then washed with PBS ($t = 24$) as above. Both parts were further processed for measurement of Pt bound to DNA. In order to decrease sample viscosity for Pt and DNA measurements, DNA was digested by nuclease P1 (7.8 μg $10^{-6}$ cells) and DNAse I (8.4 U $10^{-6}$ cells) in a buffer containing 10 mM Tris, 4 mM magnesium chloride, 0.1 mM EDTA and 0.24 mM zinc sulphate. Pt amount in these samples was measured as described previously (Hospers *et al.*, 1988) with in addition Zeeman background correction. The addition of this equipment to the AAS apparatus allowed the differential correction of the background absorption over the temperature range used to atomise the Pt, this in contrast to the fixed background value, established at one temperature, obtained without the use of Zeeman correction. Absolute values of Pt bound to DNA after 24 h were corrected for DNA synthesis over this time course, by determination of the dilution of the specific activity of DNA of cells incubated with [$^3$H]thymidine before CDDP incubation (Hospers et al., 1988). The dilution factor was calculated as: specific activity at $t = 24$ divided by specific activity at $t = 0$. Immediately after all CDDP incubations and at $t = 24$ in the repair samples, cell viability was tested by trypan blue dye exclusion.

Differences between the group of germ cell tumour and the group of colon carcinoma cell lines were analysed with the Student's t-test for unpaired samples. Correlation coefficients were determined by Spearman rank analyses. Analyses of variance were performed using the SPSS program for medical statistics (SPSS, Chicago, MI, USA). Only P-values $<0.05$ were considered significant.

### Results

The survival curves of the cell lines after continuous CDDP incubation are shown in Figure 1. The CDDP concentrations inhibiting survival by 50% ($IC_{50}$) ranged from 0.69 to 7.90 µM, thus a maximum 11.4-fold difference in sensitivity between the cell lines was found. The germ cell tumour cell lines are the most sensitive lines with $IC_{50}$ values ranging from 0.69 to 2.42 µM, while the $IC_{50}$ values of the colon carcinoma cell lines ranged from 4.16 to 7.90 µM. There was a significant difference between the mean $IC_{50}$ of the germ cell tumour and the colon carcinoma cell lines ($P<0.01$).

**Figure 1** Survival of Tera (○), 833 KE (□), Scha (△), Colo 320 (●), SW 948 (■) and Caco-2 (▲) after continuous incubation with CDDP measured by microculture tetrazolium assay. The mean $IC_{50}$ values of the group of germ cell tumour and colon carcinoma cell lines were significantly different ($P<0.01$; $n = 3$).

In Table II the basic phenotypical parameters, namely cell doubling time, relative cell volume, cellular protein, nuclear protein, cellular DNA content and percentage of cells in S-phase, are shown for all cell lines. For these characteristics up to 4-fold differences were observed. Only relative cell volume was significantly larger in the group of germ cell tumour than in the group of colon carcinoma cell lines ($P<0.025$). No correlations with CDDP sensitivity were found as far as these basic cellular characteristics were concerned.

Mean cellular GSH level (Table III) was not significantly lower in the germ cell tumour than in the colon carcinoma cell lines. But GSH levels of the six cell lines were positively correlated with CDDP $IC_{50}$ ($r = 0.90$, $P<0.05$). Surprisingly, TSH levels showed an inverse correlation with CDDP $IC_{50}$ ($r = -0.94$, $P<0.05$). There was also a significant difference between both groups of cell lines with respect to TSH ($P<0.025$) (Table III). GST activity and the amount of GSTπ (Table III) were not significantly different between both groups of tumours, nor was either parameter statistically related to CDDP sensitivity. There was no relation between GST activity and the amount of GSTπ in the cell lines.

No difference was found between the topo I activities in the nuclear protein extracts of all cell lines, and activities were similar to the topo I activity of GLC4 (for GLC4 a mean of 1 ± 1 ng nuclear protein was needed to observe complete relaxation of plasmid DNA). When topo II activity was expressed relative to GLC4 (for GLC4, with a mean of 10 ± 4 ng nuclear protein or less decatenation was no longer observed), five of the cell lines had higher topo II activities. The mean topo II activity was not different between the group of germ cell tumour and the group of colon carcinoma cell lines. Within the panel of cell lines tested, one germ cell tumour cell line had a higher topo II (833 KE) and one colon carcinoma cell line a lower topo II (Colo 320). There was no correlation between topo II activity and the amount of nuclear protein, DNA per cell or percentage of cells in S-phase.

Cellular Pt content in the various cell lines is shown in Figure 2. Immediately after 4 h incubation with CDDP no loss of cell viability was observed (data not shown). Neither a difference in accumulation between both groups nor a relation with CDDP sensitivity was found. Correlation of cellular Pt content for cellular protein or relative cell volume did not reveal a more obvious statistical relation with CDDP sensitivity. In the most sensitive germ cell tumour cell line, Tera, a low cellular Pt level was found, while in Scha cellular Pt levels were generally high compared with other cell lines. Although in two of the three germ cell carcinoma cell lines a relatively high amount of Pt bound to DNA was found (833 KE, Scha) neither a difference between the groups of tumour cell lines nor a correlation with CDDP sensitivity was found (Figure 3). Calculation of the amount of Pt bound to DNA per $10^6$ cells, thus correcting for the large differences in DNA content per cell, did not improve the relation between Pt bound to DNA and CDDP sensitivity. Measurement of Pt bound to DNA 24 h after a 4 h CDDP incubation revealed an increase, compared with $t = 0$, in one germ cell tumour and one colon carcinoma cell line, no alteration over this period in another cell line of both cell types and a decrease of Pt bound to DNA in the third cell line of both cell types

**Table II** Cellular characteristics of the three germ cell tumour (Tera, 833 KE, Scha) and the three colon carcinoma (Colo 320, SW 948, Caco-2) cell lines expressed as indicated

| Cell line | Doubling time | Relative cell volume[a] | Cellular protein (µg $10^{-6}$ cells) | Nuclear protein (µg $10^{-6}$ nuclei) | DNA content (µg $10^{-6}$ cells) | Percentage of cells in S-phase |
|---|---|---|---|---|---|---|
| Tera | 14.0 ± 0.6[b] | 116.0 | 233 ± 62 | 29.1 ± 5.2 | 15.8 ± 4.5 | 46.7 ± 2.2 |
| 833 KE | 25.3 ± 2.1 | 110.0 ± 5.8 | 372 ± 43 | 49.4 ± 8.4 | 19.8 ± 3.1 | 55.1 ± 2.9 |
| Scha | 35.6 ± 11.6 | 99.6 ± 3.6 | 165 ± 18 | 52.5 ± 16.1 | 15.7 ± 0.8 | 24.6 ± 1.4 |
| Colo 320 | 15.2 ± 0.6 | 84.2 ± 1.6 | 235 ± 12 | 26.9 ± 3.7 | 7.6 ± 0.8 | 42.7 ± 2.3 |
| SW 948 | 19.0 ± 1.2 | 76.8 ± 2.4 | 356 ± 45 | 30.8 ± 7.4 | 25.7 ± 2.4 | 56.7 ± 3.2 |
| Caco-2 | 30.3 ± 11.4 | 91.0 ± 1.2 | 330 ± 30 | 95.6 ± 10.2 | 31.8 ± 4.7 | 31.0 ± 10.0 |

[a]Significant difference between the group of germ cell and the group of colon carcinomas ($P<0.025$). [b]Mean ± s.d. ($n \geqslant 3$).

**Table III** CDDP sensitivity-related parameters of the three germ cell tumour (TERA, 833 KE, Scha) and the three colon carcinoma (Colo 320, SW 948, Caco-2) cell lines expressed per mg of cellular protein

| | *TSH* (*μg TSH*[a]) | *GSH* (*μg GSH*[b]) | *GST activity* (*nmol CDNB*[c] $min^{-1}$) | *GST-π* (*μg GST-π*) | *Topo II*[d] |
|---|---|---|---|---|---|
| Tera | 65.7 ± 10.1[e] | 1.9 ± 0.2 | 95 ± 8 | 6.3 ± 1.8 | 3 |
| 833 KE | 52.5 ± 16.9 | 5.6 ± 1.9 | 90 ± 12 | 3.9 ± 0.6 | 10 |
| Scha | 38.6 ± 4.0 | 9.8 ± 1.6 | 36 ± 9 | 6.9 ± 1.2 | 3 |
| Colo 320 | 25.7 ± 8.3 | 7.7 ± 0.8 | 75 ± 40 | 4.2 ± 0.4 | 1 |
| SW 948 | 26.5 ± 9.9 | 9.8 ± 2.9 | 68 ± 31 | 4.3 ± 0.5 | 3 |
| Caco-2 | 21.4 ± 6.0 | 12.3 ± 1.8 | 255 ± 25 | 7.2 ± 0.9 | 3 |

[a]Spearman rank analysis of TSH vs $IC_{50}$, $r = -0.94$, $P < 0.05$. [b]Spearman rank analysis of GSH vs $IC_{50}$, $r = 0.90$, $P < 0.05$. [c]1-Chloro-2,4-dinitrobenzene. [d]Topo II activity relative to the cell line GLC4 (de Jong *et al.*, 1990). Topo II activity for GLC4 was no longer seen when 10 ± 4 ng protein per lane or less was used. [e]Mean ± s.d., ($n \geq 3$).

**Figure 2** Cellular Pt accumulation after 4 h CDDP incubation determined with AAS of Tera (1), 833 KE (2), Scha (3), Colo 320 (4), SW 948 (5) and Caco-2 (6) ($n = 3-4$). Neither a significant difference between both groups of cell lines nor a correlation of Pt accumulation with CDDP sensitivity was found.

**Figure 3** Amount of Pt bound to DNA after 4 h CDDP incubation determined with AAS of Tera (1), 833 KE (2), Scha (3), Colo 320 (4), SW 948 (5) and Caco-2 (6) ($n = 4$). Neither a significant difference between both groups of cell lines nor a correlation of Pt bound to DNA with CDDP sensitivity was found.

(Table IV). Values shown in Table IV are corrected for DNA synthesis. DNA specific activity decreased; dilution factors varied from 0.46 to 0.74 in the six cell lines and were not different between the group of germ cell tumour and the group of colon carcinoma cell lines. Although within the group of germ cell tumour cell lines a good correlation between Pt bound to DNA at $t = 24$ and $IC_{50}$ was found, no correlation was found within the group of colon carcinoma cell lines.

Statistical processing of data by analysis of variance was performed to detect whether it was possible to relate combinations of parameters to sensitivity. The sensitivity of the cell lines, defined as CDDP $IC_{50}$, was correlated with GSH in combination with Pt bound to DNA (100 μM CDDP incubation) and GST activity ($r = 0.997$, $P = 0.010$). Any other combination of parameters did not reveal significant correlations with $IC_{50}$. However, even for a combination of parameters, no difference between the group of germ cell tumour and the group of colon carcinoma cell lines was found at the $IC_{50}$ level. Analyses of variance were also performed in relation to a level of a CDDP concentration inhibiting survival by 10% ($IC_{10}$) and a concentration inhibiting survival by 90% ($IC_{90}$). $IC_{10}$ correlated with GSH combined with cellular Pt accumulation (67 μM CDDP incubation) and topo II activity ($r = 0.994$, $P = 0.018$). When $IC_{10}$ was related to the combination of GSH/Pt accumulation there was also a significant difference between both groups of cell lines. When $IC_{90}$ values were correlated with Pt bound to DNA (100 μM CDDP incubation) and TSH, a difference between the groups of cell lines was found as well as correlation with sensitivity at this high cytotoxicity level.

### Discussion

In this study the survival curves of the cell lines after CDDP incubation show that the model with germ cell tumour and colon carcinoma cell lines as representatives of CDDP-

Table IV Values of Pt bound to DNA immediately ($t = 0$) and 24 h ($t = 24$) after a 4 h incubation with 16.5 μM CDDP for the three germ cell tumour (Tera, 833 KE, Scha) and 33 μM for the three colon carcinoma (Colo 320, SW 948, Caco-2) cell lines

| | *Pt bound to DNA*, t = *0 h* (*ng mg*$^{-1}$ *DNA*) | *Pt bound to DNA*, t = *24 h* (*ng mg*$^{-1}$ *DNA*) |
|---|---|---|
| Tera | 28.2 ± 19.7[a] | 61.3 ± 43.3 |
| 833 KE | 32.1 ± 6.5 | 35.2 ± 23.3 |
| Scha | 26.9 ± 4.2 | 16.6 ± 3.2 |
| Colo 320 | 48.4 ± 10.2 | 32.2 ± 15.7 |
| SW 948 | 46.3 ± 12.8 | 61.7 ± 26.0 |
| Caco-2 | 48.7 ± 3.3 | 44.0 ± 8.1 |

[a]Mean ± s.d. ($n = 3-4$).

sensitive and -resistant tumours mimics the clinical situation. The advantage of this model is that it allows the analysis of parameters of possible relevance to CDDP sensitivity in intrinsically resistant cells, in which different mechanisms may be operational than those activated in acquired resistance. Analogous models have been described previously (Bedford *et al.*, 1988; Fry *et al.*, 1991; McLaughlin *et al.*, 1993). Germ cell tumour cell lines (sensitive) have been compared with bladder carcinoma cell lines (resistant) with respect to topo II levels and sensitivity to drugs that exert activity via topo II (Fry *et al.*, 1991). Also, the correlation of CDDP sensitivity with the capacity to repair specific Pt-DNA adducts of germ cell and bladder tumour cell lines has been described (Bedford *et al.*, 1988). In another study the binding activities of cisplatin damage recognition proteins in germ cell and bladder tumour cell lines were compared (McLaughlin *et al.*, 1993). In the present study the basic characteristics of the cell lines and multiple mechanisms previously described to be of importance in *in vitro* acquired resistance were evaluated including topo II activity and the kinetics of Pt bound to DNA. Basic characteristics varied widely among the six cell lines. But correction of resistance-related parameters for differences in basic characteristics that were thought to affect the estimation of a certain resistance mechanism did not influence statistical correlations.

Increased GSH is generally considered a relevant mechanism in CDDP resistance (Hosking *et al.*, 1990; Meijer *et al.*, 1990; Mistry *et al.*, 1991). It has been described to play a role in germ cell tumour (Meijer *et al.*, 1992; Timmer-Bosscha *et al.*, 1993) as well as in colon carcinoma cell lines (Fram *et al.*, 1990) with acquired CDDP resistance. In our panel of cell lines cellular GSH content showed a positive correlation with $IC_{50}$. This correlation with a median cytotoxicity level differed from the results obtained by Peters *et al.* (1991). They found that GSH protection was most effective at CDDP concentrations inducing over 90% kill when they compared GSH-depleted with wild-type K562 cells. Our findings might indicate that at higher CDDP cytotoxicity levels the protective potential of GSH is overwhelmed by the extensive cellular damage caused by CDDP. TSH consists of GSH and protein-bound sulphydryl groups. Protein-bound sulphydryl is usually thought to be indicative of the amount of cellular metallothionein, a group of proteins with a high cysteine content. Elevated amounts of metallothionein are found in some cell lines with acquired CDDP resistance (for review see Andrews and Howell, 1990). But the role of metallothioneins in the CDDP sensitivity of tumour cells has been mainly established in cell lines in which metallothionein has been induced with other heavy metals (for review see Andrews and Howell, 1990). However, the role of metallothioneins in CDDP resistance seems complex. Recently, it has been reported that in Chinese hamster ovary cells transient induction of constitutive metallothionein leads to decreased CDDP sensitivity (Koropatnick and Pearson, 1993). In contrast, in the same cell line, overexpression of a transfected mouse metallothionein gene leads to an increased CDDP sensitivity (Koropatnick and Pearson, 1993). In the present model higher amounts of TSH were found in the germ cell tumour cell lines than in the colon carcinoma lines. In view of the data of Koropatnick and Pearson (1993), this should imply that the metallothioneins in the present model behaved like the transfected mouse metallothioneins and not like those induced by heavy metal incubation of the cells. Another possible explanation might be the finding that rat testicular metal-binding proteins were not, in contrast to the metal-binding proteins in other tissues, metallothioneins (Waalkes and Perantoni, 1986). Compared with metallothionein, these testicular proteins had a similar molecular weight but a different amino acid composition; most striking was the small amount of cysteine residues present. Analogously, it could be that the sulphydryl-containing proteins in germ cell tumours were different from those in colon carcinoma. Based on the inverse correlation between TSH and $IC_{50}$, it could even be speculated that, if these proteins do bind Pt, a toxic complex might be formed in the germ cell tumour cell lines. Increased GST activity has been found in several cell lines with acquired CDDP resistance (for review see Andrews and Howell, 1990). However, in our study no direct correlation of GST activity with CDDP sensitivity was observed. In the analysis of variance, GST activity in combination with GSH level and Pt bound to DNA showed a correlation with CDDP sensitivity at $IC_{50}$. This is compatible with a role of GST in cellular detoxification in these cell lines. There was no difference between both groups of cell lines with respect to GST activity of amount of GSTπ. However, the GST activity in colon carcinomas is generally reported to be increased (Moscow *et al.*, 1989; Peters *et al.*, 1992), while in germ cell tumours GST activity is decreased compared with normal adjacent tissue (Strohmeyer *et al.*, 1992). Probably normal germ cells depend on a high GST level for cellular detoxification and, therefore, the lower GST in the germ cell tumour cell lines may be one of the causes of their CDDP sensitivity.

Topo I activity was similar in all cell lines. Giaccone *et al.* (1992) also described only small differences in topo I RNA expression in *in vitro* untreated lung carcinoma cell lines, while in these lines CDDP sensitivity varied as well as sensitivity to drugs for which topo I is the target. Also, in an ovarian carcinoma cell line with *in vitro* acquired CDDP resistance, cross-resistance for a topo I-directed drug in the absence of a difference in topo I levels was found (Niimi *et al.*, 1992). So, although it cannot be stated on the basis of these results that topo I does not play a role in CDDP resistance, its activity is not a predictive factor for CDDP sensitivity *in vitro*. Fry *et al.* (1991) found a higher topo II expression in germ cell tumour cell lines than in bladder tumour cell lines, and in a panel of lung cancer cell lines high topo II RNA expression correlated with sensitivity for multiple drugs, including CDDP (Giaccone *et al.*, 1992). In cell lines with acquired CDDP resistance decreased (Yang and Douple, 1991) as well as increased (De Jong *et al.*, 1991) topo II activities have been found compared with their sensitive parental lines. In our cell lines we found no correlation between topo II activity and CDDP sensitivity and no difference between the two groups of tumour types. The high topo II activity in 833 KE was in agreement with the high levels in this cell line described by Fry *et al.* (1991). But a high topo II level was not found in the two other germ cell tumour cell lines. Combined with the varying results that have been found in acquired CDDP resistance, these data exclude a direct, common role for topo II in CDDP resistance.

Decrease of cellular Pt uptake in cells with *in vitro* acquired resistance is a frequent mechanism of resistance (for review see Gately and Howell, 1993), and the amount of Pt bound to DNA in tumour cells *in vitro* is often correlated with CDDP sensitivity (Sherman and Lippard, 1987). In a report by Bedford *et al.* (1988), accumulation of Pt in two germ cell tumour and one bladder tumour cell line correlated with CDDP sensitivity. The amount of initial DNA platination after 1 h incubation with CDDP was not in agreement with the CDDP sensitivity of these lines. However, the amount of Pt bound to DNA after 1 h incubation with

CDDP followed by 24 h culture offered a good correlation (Bedford *et al.*, 1988). In contrast, Hill *et al.* (1994) found the lowest DNA platination in the most sensitive lines and the highest in the most insensitive lines, using a panel of four germ cell tumour cell lines and an incubation and post-incubation scheme according to Bedford *et al.* (1988). In our model cellular Pt uptake did not correlate with sensitivity. The germ cell tumour cell line Scha, for example, had a high cellular Pt level, while the most sensitive germ cell tumour cell line, Tera, had a low cellular Pt content. Pt bound to DNA did not correlate with sensitivity unless combined with parameters of cellular detoxification. This is surprising as Pt binding to DNA is usually considered to be the mechanism of toxicity of CDDP. Correlation with Pt bound to DNA at $t = 24$ h was found in the group of germ cell tumour cell lines, but not in the group of colon carcinoma cell lines. An increase in Pt-DNA levels during the 24-h post-incubation period was also found in two of the germ cell tumour cell lines described by Hill *et al.* (1994). As that study used an immunochemical technique whereas the present study used AAS to measure Pt-DNA, it is unlikely that this increase is due to an artifact of the technique used. The fact that we also found increases in Pt bound to DNA in one colon carcinoma and no repair in another seemed contrary to the results reported by Bedford *et al.* (1988). In that study the resistant cell line was incubated with a concentration of CDDP close to its own $IC_{50}$. In our study the resistant cell lines were incubated with CDDP concentrations exceeding their $IC_{50}$ by 4- to 8-fold. In these circumstances a lack of repair capacity is also found in the colon carcinoma cell lines. This suggested that the lack of repair capacity described for germ cell tumour cell lines (Bedford *et al.*, 1988; Kelland *et al.*, 1992; Hill *et al.*, 1994) might be at least partly due to the high incubation concentrations relative to their $IC_{50}$ values in the studies described, and not only to a unique phenomenon in germ cell tumour cell lines.

Analysis of variance was used to correlate combinations of parameters with sensitivity. It showed a good correlation between $IC_{50}$, the parameter normally used to indicate drug sensitivity, and Pt bound to DNA in combination with GSH and GST activity. However, at other cytotoxicity levels ($IC_{10}$ and $IC_{90}$) also, combined correlations with other parameters such as topo II activity, cellular Pt content and TSH were found. The diversity of correlations might indicate that in these cell lines different mechanisms may be important at different levels of sensitivity, although since only a few cell lines were analysed the results can only be used as a guideline. The fact that at all cytotoxicity levels one or more components of the detoxifying system are involved is a strong indication for the relevance of this system in intrinsic tumour cell CDDP sensitivity. On the other hand, the contribution of a certain parameter in the various cell lines might vary widely. For instance, in the germ cell tumour cell line Scha, the GSH level was similar to that in the colon carcinoma cell line SW 948. But the high Pt accumulation in Scha seemed to overwhelm its GSH pool, leading to a higher Pt-DNA binding ($t = 0$) in Scha than in SW 948 after incubation with the same CDDP concentration. The colon carcinoma cell line Caco-2 exhibited a high degree of Pt accumulation but a low level of Pt-DNA binding. This might be caused by a high efficacy of its detoxifying system, as GSH levels and GST activity were the highest in this cell line.

Based on our model of unselected cell lines it can be concluded that multiple parameters must be analysed to explain CDDP resistance *in vitro*. However, other parameters that we did not include in this study (for review see Kelland, 1994) might explain the sensitivity of germ cell tumour and the insensitivity of colon carcinoma cell lines. Thus the sensitivity of germ cell tumours to a range of drugs inducing DNA damage (Masters *et al.*, 1993) and the possible role of DNA damage recognition proteins (McLaughlin *et al.*, 1993) are intriguing.

In conclusion, the study described here did not reveal a cause of the unique sensitivity of germ cell tumours, but the unexpected relation between $IC_{50}$ and TSH should be studied further.

**Abbreviations**: AAS, atomic absorption spectrophotometry; CDDP, cisplatin, *cis*-diamminedichloroplatinum(II); CDNB, 1-chloro-2,4-dinitrobenzene; FCS, fetal calf serum; GSH, glutathione; GST, glutathione *S*-transferase; $IC_{10}$, drug concentration inhibiting survival by 10%; $IC_{50}$, drug concentration inhibiting survival by 50%; $IC_{90}$, drug concentration inhibiting survival by 90%; PBS, phosphate-buffered saline (0.14 M sodium chloride, 2.7 mM potassium chloride, 6.4 mM disodium hydrogen phosphate, 1.5 mM potassium dihydrogen phosphate, pH 7.4); Pt, platinum; topo, DNA topoisomerase; TSH, total sulphydryl content.

**Acknowledgements**
The authors would like to thank Tineke van der Sluis for technical assistance. This study was supported by a grant from the Dutch Cancer Foundation (GUKC 90-18).

### References

ANDREWS PA AND HOWELL SB. (1990). Cellular pharmacology of cisplatin: perspectives on mechanisms of acquired resistance. *Cancer Cells*, **2**, 35-43.

ANDREWS PW, OOSTERHUIS JW AND DAMJANOV I. (1987). Cell lines from human germ cell tumors. In *Teratocarcinomas and Embryonic Stem Cell Lines*, Robertson E (ed.) pp. 207-248. IRL Press: Oxford.

BEDFORD P, FICHTINGER-SCHEPMAN AMJ, SHELLARD SA, WALKER C, MASTERS JRW AND HILL BT. (1988). Differential repair of platinum-DNA adducts in human bladder and testicular tumour continuous cell lines. *Cancer Res.*, **48**, 3019-3024.

BRADFORD MM. (1976). A rapid and sensitive method for the quantitation of microgram quantities of protein utilizing the principle of protein dye binding. *Anal. Biochem.*, **72**, 248-254.

BRONSON DL, ANDREWS PW, SOLTER D, CEREVENKA J, LANGE PH AND FRALEY EE. (1980). Cell line derived from a metastasis of a human testicular germ cell tumour. *Cancer Res.*, **40**, 2500-2506.

DE JONG S, ZIJLSTRA JG, DE VRIES EGE AND MULDER NH. (1990). Reduced DNA topoisomerase II activity and drug-induced DNA cleavage activity in an adriamycin-resistant human small cell lung carcinoma cell line. *Cancer Res.*, **50**, 304-309.

DE JONG S, ZIJLSTRA JG, MULDER NH AND DE VRIES EGE. (1991). Lack of cross-resistance to fostriecin in a human small-cell lung carcinoma cell line showing topoisomerase II-related drug resistance. *Cancer Chemother. Pharmacol.*, **28**, 461-464.

FOGH J. (1978). Cultivation, characterization, and identification of human tumor cells with emphasis on kidney, testis and bladder tumors. *Natl Cancer Inst. Monogr.*, **49**, 5-9.

FOGH J, WRIGHT WC AND LOVELESS JD. (1977). Absence of Hela contamination in 169 cell lines derived from human tumors. *J. Natl Cancer Inst.*, **58**, 209-214.

FRAM RJ, WODA BA, WILSON JM AND ROBICHAUD NA. (1990). Characterization of acquired resistance to cis-diamminedichloroplatinum (II) in BE human colon carcinoma cells. *Cancer Res.*, **50**, 72-77.

FRY AM, CHRESTA CM, DAVIES SM, WALKER MC, HARRIS AL, HARTLEY JA, MASTERS JRW AND HICKSON ID. (1991). Relationship between topoisomerase II level and chemosensitivity in human tumor cell lines. *Cancer Res.*, **51**, 6592-6595.

GATELY DP AND HOWELL SB. (1993). Cellular accumulation of the anticancer agent cisplatin: a review. *Br. J. Cancer*, **67**, 1161-1166.

GIACCONE G, GAZDAR AF, BECK H, ZUNINO F AND CAPRANICO G. (1992). Multidrug sensitivity phenotype of human lung cancer cells associated with topoisomerase II expression. *Cancer Res.*, **52**, 1666-1674.

HILL BT, SCANLON KJ, HANSSON J, HARSTRICK A, PERA M, FICHTINGER-SCHEPMAN AMJ AND SHELLARD SA. (1994). Deficient repair of cisplatin-DNA adducts identified in human testicular teratoma cell lines established from tumours from untreated patients. *Eur. J. Cancer*, **30A**, 832-837.

HOSKING LK, WHELAN RDH, SHELLARD SA, BEDFORD P AND HILL BT. (1990). An evaluation of the role of glutathione and its associated enzymes in the expression of differential sensitivities to antitumour agents shown by a range of human tumour cell lines. *Biochem. Pharmacol.*, **40**, 1833–1842.

HOSPERS GAP, MULDER NH, DE JONG B, DE LEIJ L, UGES DRA, FICHTINGER-SCHEPMAN AMJ AND DE VRIES EGE. (1988). Characterization of a human small cell lung carcinoma cell line with acquired resistance to cisdiamminedichloroplatinum(II) in vitro. *Cancer Res.*, **48**, 6803–6807.

KELLAND LR. (1994). The molecular basis of cisplatin sensitivity/resistance. *Eur. J. Cancer*, **30A**, 725–727.

KELLAND LR, MISTRY P, ABEL G, FREIDLOS F, LOH SY, ROBERTS JJ AND HARRAP KR. (1992). Establishment and characterization of an in vitro model of acquired resistance to cisplatin in a human testicular nonseminomatous germ cell line. *Cancer Res.*, **52**, 1710–1716.

KISSANE JM AND ROBINS E. (1958). The fluorometric measurement of deoxyribonucleic acid in animal tissues with special reference to the central nervous system. *J. Biol. Chem.*, **233**, 184–188.

KOROPATNICK J AND PEARSON J. (1993). Altered cisplatin and cadmium resistance and cell survival in chinese hamster ovary cells expressing mouse metallothionein. *Mol. Pharmacol.*, **44**, 44–50.

LEIBOVITZ A, STINSON JC, MCCOMBS III WB, MCCOY CE, MAZAR KC AND MABRY ND. (1976). Classification of human colorectal adenocarcinoma cell lines. *Cancer Res.*, **36**, 4562–4569.

LOEHRER PJ AND EINHORN LH. (1984). Cisplatin. *Ann. Intern. Med.*, **100**, 704–713.

LOWRY OH, ROSENBROUGH NJ, FARR AL AND RANDALL RJ. (1951). Protein measurement with the folin-phenol reagent. *J. Biol. Chem.*, **193**, 265–276.

MCLAUGHLIN K, COREN G, MASTERS J AND BROWN R. (1993). Binding activities of cisplatin-damage-recognition proteins in human tumour cell lines. *Int. J. Cancer*, **53**, 662–666.

MASTERS JRW, OSBORNE EJ, WALKER MC AND PARRIS CN. (1993). Hypersensitivity of human testis tumour cell lines to chemotherapeutic drugs. *Int. J. Cancer*, **53**, 340–346.

MEIJER C, MULDER NH AND DE VRIES EGE. (1990). The role of detoxifying systems in resistance of tumour cells to cisplatin and adriamycin. *Cancer Treat. Rev.*, **16**, 389–407.

MEIJER C, MULDER NH, TIMMER-BOSSCHA H, SLUITER WJ, MEERSMA GJ AND DE VRIES EGE. (1992). Relationship of cellular glutathione to the cytotoxicity and resistance of seven platinum compounds. *Cancer Res.*, **52**, 6885–6889.

MISTRY P, KELLAND LR, ABEL G, SIDHAR S AND HARRAP KR. (1991). The relationship between glutathione, glutathione-*S*-transferase and cytotoxicity of platinum drugs and melphalan in eight human ovarian carcinoma cell lines. *Br. J. Cancer*, **64**, 215–220.

MOSCOW JA, FAIRCHILD CR, MADDEN MJ, RANSOM DT, WIEAND HS, O'BRIEN EE, POPLACK DG, COSSMAN J, MYERS CE AND COWAN KH. (1989). Expression of anionic glutathione S-transferase and P-glycoprotein genes in human tissues and tumors. *Cancer Res.*, **49**, 1422–1428.

NIIMI S, NAKAGAWA K, SUGIMOTO U, NISHIO K, FUJIWARA Y, YOKOYAMA S, TERASHIMA U AND SAIJO N. (1992). Mechanisms of cross-resistance to a camptotecin analogue (CPT-11) in a human ovarian cancer cell line selected by cisplatin. *Cancer Res.*, **52**, 328–333.

OOSTERHUIS JW, ANDREWS PW, KNOWLESS BB AND DAMJANOV I. (1984). Effects of cisplatinum on embryonal carcinoma cell lines in vitro. *Int. J. Cancer*, **34**, 133–139.

PARRIS CN, WALKER MC, MASTERS JRW AND ARLETT CF. (1990). Inherent sensitivity and induced resistance to chemotherapeutic drugs and irradiation in human cancer cell lines: relationship to mutation frequencies. *Cancer Res.*, **50**, 7513–7518.

PERA MF, FRIEDLOS F, MILLS J AND ROBERTS JJ. (1987). Inherent sensitivity of cultured human embryonal carcinoma cells to adducts of cis-diamminedichloroplatinum (II) on DNA. *Cancer Res.*, **47**, 6810–6813.

PETERS RH, JOLLOW DJ AND STUART RK. (1991). Role of glutathione in the in vitro synergism between 4-hydroperoxy cyclophosphamide and cisplatin in leukemia cell lines. *Cancer Res.*, **51**, 2536–2541.

PETERS WHM, NAGENGAST FM AND WOBBES T. (1989). Glutathione *S*-transferases in normal and cancerous human colon tissue. *Carcinogenesis*, **12**, 2371–2374.

PETERS WHM, BOON CEW, ROELOFS HMJ, WOBBES T, NAGENGAST FM AND KREMERS PG. (1992). Expression of drug-metabolizing enzymes and P-160 glycoprotein in colorectal carcinoma and normal mucosa. *Gastroenterology*, **103**, 448–455.

PREISLER HD, GOPAL V, BANAVALI SD, FINKE D AND BOKARI SAJ. (1992). Multiparameter assessment of the cell cycle effects of bioactive and cytotoxic agents. *Cancer Res.*, **52**, 4090–4095.

QUIN LA, MORE GE, MORGAN RT AND WOODS LK. (1979). Cell lines from human colon carcinoma with unusual cell products, double minutes and homogeneously staining regions. *Cancer Res.*, **39**, 4914–4924.

SHERMAN SE AND LIPPARD SJ. (1987). Structural aspects of platinum anticancer drug interaction with DNA. *Chem. Rev.*, **87**, 1153–1181.

STROHMEYER T, KLÖNE A, WAGNER G, HARTMANN M AND SIES H. (1992). Glutathione S-transferase in human testicular germ cell tumors: changes of expression and activity. *J. Urol.*, **147**, 1424–1428.

TIMMER-BOSSCHA H, HOSPERS GAP, MEIJER C, MULDER NH, MUSKIET FAJ, MARTINI IA, UGES DRA AND DE VRIES EGE. (1989). Influence of docosahexaenoic acid on cisplatin resistance in a human small cell lung carcinoma cell line. *J. Natl Cancer Inst.*, **81**, 1069–1075.

TIMMER-BOSSCHA H, TIMMER A, MEIJER C, DE VRIES EGE, DE JONG B, OOSTERHUIS JW AND MULDER NH. (1993). Cisdiamminedichloroplatinum(II) resistance in vitro and in vivo in human embryonal carcinoma cells. *Cancer Res.*, **53**, 5707–5713.

WAALKES MP AND PERANTONI A. (1986). Isolation of a novel metal-binding protein from rat testes. *J. Biol. Chem.*, **261**, 13097–13103.

WALKER MC, PARRIS CN AND MASTERS JRW. (1987). Differential sensitivities of human testicular and bladder tumor cell lines to chemotherapeutic drugs. *J. Natl Cancer Inst.*, **79**, 213–216.

YANG LX AND DOUPLE EB. (1991). Correlations of topoisomerase II levels and activity with cisplatin resistance and sensitivity in Walker tumour cell lines. *Proceedings of the Sixth International Symposium on Platinum and other Metal Coordination Compounds in Cancer Chemotherapy, Vol. 103.*